# Progression of lumbar disc herniations over an eight-year period in a group of adult Danes from the general population – a longitudinal MRI study using quantitative measures

**DOI:** 10.1186/s12891-016-0865-6

**Published:** 2016-01-15

**Authors:** Per Kjaer, Andreas Tunset, Eleanor Boyle, Tue Secher Jensen

**Affiliations:** Department of Sports Science and Clinical Biomechanics, University of Southern Denmark, Campusvej 55, Odense M, DK-5230 Denmark; Dalla Lana School of Public Health, University of Toronto, 155 College Street, 6th floor, Toronto, ON M5T 3M7 Canada; Medical Department, Spine Centre of Southern Denmark, Lillebaelt Hospital, Oestre Hougvej 55, Middelfart, DK-5500 Denmark; Institute of Regional Health Research, University of Southern Denmark, Campusvej 55, Odense M, DK-5230 Denmark

**Keywords:** Magnetic resonance imaging, Intervertebral disc, Disc herniation, Lumbar spine, Longitudinal development, Disc degeneration, Quantitative measurements, Spinal canal, Dural sac, Disc height

## Abstract

**Background:**

A lumbar disc herniation (LDH) is a localised displacement of disc material, which may initiate changes in the disc and adjacent structures such as the nerve root and the spinal canal. Knowledge about how morphological changes in the disc relate to changes in other spinal structures might give the clinician a better understanding of the natural history and consequences of lumbar disc herniations. However, few longitudinal studies have investigated this process using reliable measures from magnetic resonance imaging (MRI). The objectives of this study were to examine changes in and associations between the size of lumbar disc herniations, dural sac area and disc height over an eight-year period using MRI at three time-points.

**Methods:**

Individuals from a population-based cohort, the ‘Backs on Funen Cohort’, had MRIs taken at age 41 years and again at 45 and 49 years. Only disc levels with MRI-confirmed disc herniations at 41 or 45 years were included. Cross-sectional areas (mm^2^) of the LDH, dural sac and disc height were calculated from measurements performed on sagittal T2-weighted images using a previously validated method. Changes over time for the three MRI findings were defined as “unchanged”, “increased “, “decreased”, or “fluctuating”. Only changes beyond 95 % limits of agreement of the same measurements were regarded as valid. Associations between the three types of measures were examined cross-sectionally and longitudinally.

**Results:**

One hundred and forty disc levels, from 106 people (48 women and 58 men), were included. Over eight years, 65 % of the herniations remained unchanged, 17.5 % decreased, 12.5 % increased, and 5 % had a fluctuating pattern. Increased herniation size was associated with decreased dural sac area (β-0.25[−0.52;0.01]) and increased disc height (β 0.35[0.14;0.56]). Moreover, larger herniation size predicted a statistically significant reduction in both dural sac area (β-0.35[−0.58;-0.13]) and disc height (β-0.50[−0.81;-0.20]).

**Conclusions:**

On average, most LDHs do not change over a four- to eight-year period. However, larger herniation size predicts a reduction in both dural sac area and disc height. Further research should be done to determine the correlations between the progression of LDH and resolution of patient symptoms.

## Background

Lumbar disc herniations (LDH) are defined as localised displacements of disc material beyond the limits of the intervertebral disc space [1]. They are typically classified qualitatively from the morphologic appearance of disc contour on magnetic resonance imaging (MRI) into protrusion, extrusion and sequestration [1].

The long-term changes for LDH, as well as the processes leading to such changes, are not well understood. A recent systematic review of longitudinal studies of patients with sciatica, receiving conservative treatment, reported that regression of LDH is seen in 30 % of protrusions and 77 % of extrusions and sequestrations [2]. Another recent study, not included in that review, showed a decrease in LDH size in half of the cases, and an increase in size in the other third [3].

The majority of those studies used only a single follow up and none of them had MRI follow up of all included patients for more than 2 years [2, 3].

The way in which LDH affects the intervertebral disc height and the dural sac is sparsely described in the literature [4]. It has been hypothesised that disc height is influenced by nucleus material being displaced through annular fissures to the periphery or external to the disc causing the disc to collapse [5, 6], although this assumption has been challenged [7]. Following this line of thought, there could be an association between LDH and the dural sac area, where disc material is displaced posteriorly into the spinal canal resulting also in a reduction in disc height [8, 9]. Based on this, we hypothesised that an increase in the size of LDH leads to a decrease in disc height and dural sac size over time.

Therefore, the specific objectives of this study were to: (1) describe changes in the size of LHD, dural sac and disc height over an eight-year period in discs with LDH, and (2) quantify any cross-sectional and/or longitudinal associations between the three MRI findings.

## Methods

### Design

The study was a longitudinal population-based observational study.

### Study population and material

This study used the magnetic resonance images from the Danish longitudinal cohort-study, ‘Backs on Funen’, which investigated potential risk factors of low back pain (LBP) in a general population [10]. In brief, the Office of Civil Registrations generated a sample from all Danes aged 40 years in 2000 and living in the county of Funen, an island in Denmark with about 450,000 inhabitants. An invitation letter was mailed to a random sample of 11 % of these 40-year-olds, corresponding to 625 people. Reasons for exclusion were severe disability, ferromagnetic implants, claustrophobia or inability to communicate in Danish [10]. Of these people, 412 (66 %) consented and participated in the baseline measurements (Time 1) and 48 % were male. At Time 1 they had a clinical examination, lumbar MRI and completed a questionnaire. Four years later (Time 2), 348 participants (56 %) completed the first follow-up visit (46 % were male), and another four years later (Time 3), 293 participants (47 %) completed the second follow-up visit (46 % were male). At these follow-up visits, the participants repeated both the questionnaire and lumbar MRI. Details about the socio-demographics and back pain have been published previously [11, 12]. Approximately, 70 % of the cohort reported back pain within the past year at each time point. Ethics approval was granted for the original study [10] from the Ethics Committee of Vejle and Funen Counties (approval no: 20000042) and for access to the database by the Danish Data Protection Agency (approval no: 2000-52-0037). All participants gave their informed written consent prior to study enrolment.

Lumbar disc levels with broad-based or focal protrusions, extrusion or sequestration at Time 1 or Time 2 were selected for this study according to the criteria outlined by the ‘Combined Task Force’ [13].

### MRI

MRI scans were performed with an open, low field 0.2 T magnetic resonance unit (Magnetom Open Viva, Siemens AG, Erlangen, Germany). The lower thoracic and lumbar regions were scanned with subjects in the supine position, using a body spine surface coil. Sagittal T1- and T2-weighted and axial T2-weighted MRI images were performed with axial images placed in the plane of the five lower discs. For further details, see the original study [10].

### Definition of LDH

The qualitative evaluation of LDH was made by an experienced musculoskeletal research radiologist, who demonstrated excellent reliability in the rating of disc contour in the same cohort of people [10, 14]. The intra-and inter-observer agreement for the evaluation of disc contour was substantial, kappa =0.78 ((95 % confidence intervals (CI) 0.64-0.91) for intra-observer and kappa = 0.68 (95 % CI 0.55-0.81) for inter-observer [14].

### Measures from MRI

Quantitative measures of disc height, LDH size and dural sac area were performed following a newly developed method for this purpose [15].

**Fig. 1:**
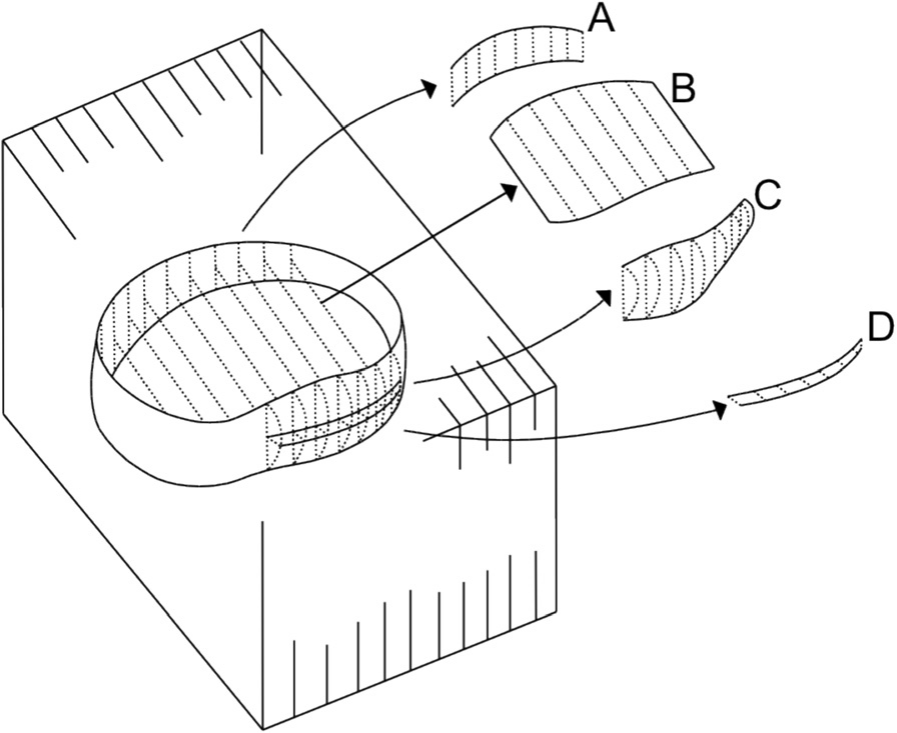
Schematic drawing of 3D cross-sectional areas (CSA) and volume of disc measures from sagittal image slices. Anterior intervertebral height (A); Intervertebral disc (B); Posterior intervertebral height (C); Posterior disc material (D). Dural sac is not shown on this figure. Figure reproduced with permission from the authors [15].

Anterior and posterior intervertebral disc heights were expressed as cross-sectional areas (CSA) calculated from measures of disc height from each sagittal image section plus the slice thickness and inter-slice gap (Fig. 1). The average disc height was calculated using the formula: (anterior intervertebral height + posterior intervertebral height)/2 [16]. LDH and dural sac CSA were also calculated using combined length measures from sagittal images and evaluated for each segment, which was given an LDH rating by the radiologist. Full details about the measurement protocol have been described in a separate manuscript [15]. The measurements were performed using the free open-source software OsiriX (version 4.1.2). This version of OsiriX is designed for scientific use [17].

All MRI measurements were conducted by a student completing a Master in Clinical Biomechanics (AT), who had previous MRI measurement experience from another study which quantified the reproducibility of the current method [15]. To ensure that the rater was blinded to participant information during measurements, all participant images were anonymised.

### Validity of measurements

The levels of intra- and inter-rater agreements of the measurements were evaluated in a previous study where they were found to be between acceptable and good [15]. The reliability of CSA calculations was also evaluated in the same study and was found to be acceptable.

### Data manipulation

Custom-made software was used to calculate length and cross-sectional areas based on the X and Y coordinates, slice thickness and inter-slice gap. The method and the software have been described in detail elsewhere [15].

### Data validation

All calculated results were validated and checked for consistency with the images of the Region Of Interest (ROI) for each measurement. All values were examined in Excel files for identification of outliers and all potential outliers were validated against the ROI files. In addition, a systematic selection of approximately every tenth participant was screened for errors using ROI files.

### Data analysis

Four-year changes were defined as changes in measurements from Time 1 to Time 2 for disc levels with LDH observed at Time 1, or from Time 2 to Time 3 for disc levels with LDH observed at Time 2. Eight-year changes were defined as changes in measurements from Time 1 to Time 3, for disc levels with LDH observed at Time 1 only.

The changes in size over time *at a group level* were summarised in tables with means and 95 % CI for LDH size, dural sac areas, and disc heights for each of the three time-points by lumbar level. Changes in size were reported for each disc level, as well as for all disc levels combined. The reporting of mean values instead of median values was chosen after testing for normal distribution.

The summary statistics for changes in size of herniations *at an individual level* were conducted producing trajectories for LDH sizes defined as ‘unchanged’, ‘increased’ or ‘decreased‘ based on Limits of Agreement (LOA) for the measurements [18]. LOA for disc height was 79.9 mm^2^, for LDH 58.9 mm^2^, and for dural sac area 69.9 mm^2^ [15]. A change was only reported if the absolute value was larger than the LOA. For the eight-year analysis covering all three time-points, an extra category, ‘fluctuating’ was added for those who did not remain in the same category throughout the time period (e.g. a pattern of unchanged between Time 1 and Time 2 and a decrease between Time 2 and Time 3 would be categorised as fluctuating).

The association between the three MRI findings was evaluated cross-sectionally and longitudinally at both the short- and long-term time-points. Disc height and dural sac area acted as dependent variables and LDH and disc height as independent variables in the longitudinal univariate and multivariable linear regression analyses. In the multivariable regression analysis, the interaction between disc height and LDH was taken into account. Results from the regression analyses were presented as beta coefficients with 95 % CI. *P*-values of 0.05 or less were considered statistically significant.

All statistical analyses were conducted using STATA statistical package version number 13.1 for Mac OS X [19].

## Results

### Study sample and material

**Fig. 2:**
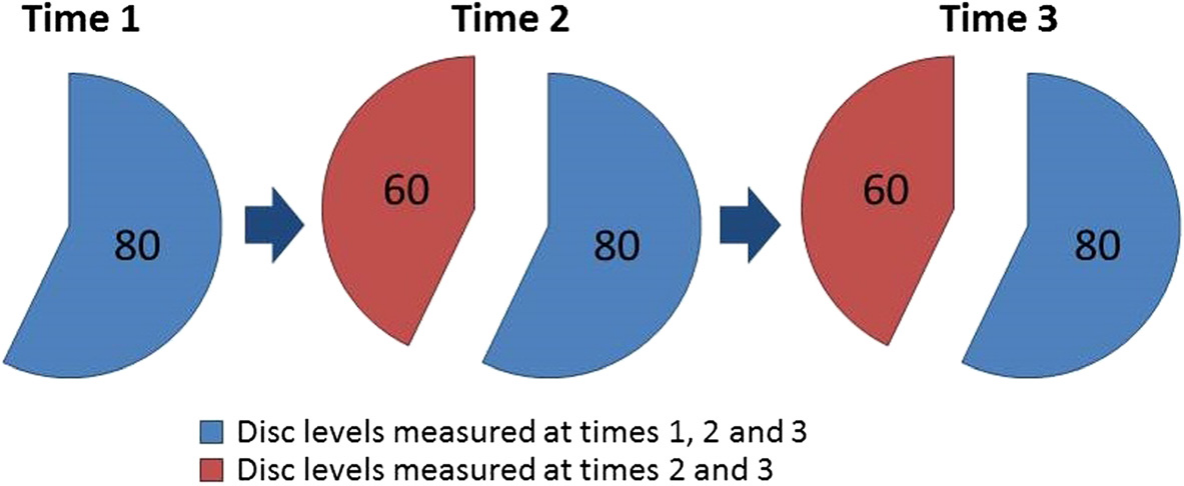
Proportion of intervertebral disc levels measured at each time-point

Based on the presence of LDH from the radiologist’s qualitative evaluation of disc contour, a total of 140 disc levels with LDH, from 106 people (48 women and 58 men) were included for measurement. Eighty disc levels with LDH were included at Time 1, and an additional 60 disc levels had developed LDH and were included at Time 2, see Fig. 2.

### Changes in size of LDH, disc heights and dural sacs

The changes in size of LDH, dural sac area and disc height at a group level over the three time-points showed no significant directional change on any of the three lowest disc levels when comparing the 95 % CIs (Tables 1 & 2).

**Table 1:** Cross-sectional areas of LDH, dural sac area, and disc height measured over three time-points (Time 1–3), presented as means with 95 % confidence intervals, for disc levels L3-L4, L4-L5, and L5-S1

Level & Time	Disc levels (n)	Mean size [95 % Confidence Intervals] (mm^2^)		
		LDH	Dural Sac	Intervertebral Height
L3-L4				
Time 1	9	132 [102; 162]	228 [187; 268]	357 [313; 401]
Time 2	9	124 [96; 152]	224 [164; 284]	350 [299; 401]
Time 3	9	125 [93; 156]	223 [161: 286]	351 [302; 399]
L4-L5				
Time 1	28	145 [133; 158]	228 [204; 252]	423 [398; 447]
Time 2	28	146 [133; 159]	214 [188; 239]	416 [393; 439]
Time 3	28	140 [126; 155]	223 [195; 252]	399 [369; 429]
L5-S1				
Time 1	43	164 [154; 174]	316 [285; 347]	437 [414; 460]
Time 2	43	166 [155; 177]	309 (276; 341]	436 [411; 461]
Time 3	43	175 [161; 189]	304 [272; 336]	421 [393; 449]

**Table 2:** Cross-sectional areas of LDH, dural sac area, and disc height measured over two time-points (Time 2–3), presented as means with 95 % confidence intervals, for disc levels L3-L4, L4-L5, and L5-S1

Level & Time	Disc levels (n)	Mean size [95 % Confidence Intervals] (mm^2^)		
		LDH	Dural Sac	Intervertebral Height
L3-L4				
Time 2	9	123 [98; 148]	252 [214; 290]	392 [337; 448]
Time 3	9	119 [94; 144]	249 [202; 295]	377 [316; 439]
L4-L5				
Time 2	23	151 [139; 162]	202 [172; 232]	431 [392; 470]
Time 3	23	147 [135; 159]	202 [170; 234]	425 [391; 459]
L5-S1				
Time 2	28	163 [148; 177]	338 [309; 368]	427 [396; 457]
Time 3	28	159 [145; 173]	336 [306; 365]	425 [395; 455]

Changes in the size of LDH, dural sac area and disc heights at each individual disc level remained ‘unchanged’ both after four and eight years for the majority of the disc levels. In general, more change was seen for all three MRI findings over the eight-year period as compared with the four-year period (Table 3).

**Table 3:** Individual trajectories for changes in size of herniations, dural sacs and disc heights shown by numbers and percentages for all time periods

Structure	Time	Number of disc levels	Decrease	Unchanged	Increase	Fluctuating
Lumbar disc herniations	Four-year change	140	19 (14 %)	114 (81 %)	7 (5 %)	-
	Eight-year change	80	14 (17,5 %)	52 (65 %)	10 (12,5 %)	4 (5 %)
Dural sac	Four-year change	140	25 (18 %)	110 (78,5 %)	5 (3,5 %)	-
	Eight-year change	80	23 (29 %)	49 (61 %)	4 (5 %)	4 (5 %)
Disc height	Four-year change	140	10 (7 %)	127 (91 %)	3 (2 %)	-
	Eight-year change	80	13 (16 %)	62 (78 %)	4 (5 %)	1 (1 %)

Total measurements of disc levels for each time category included. Missing values for “fluctuating” are due to the use of only one time period

### Associations between intervertebral disc structures and dural sac area

There was a statistically significant association between LDH and disc height in the cross-sectional, four-year and eight-year analyses (*p* < 0.001, Table 4). Cross-sectionally, an increment of 100 mm^2^ in herniation area was associated with an increase in disc height area of 35 mm^2^. For each 100 mm^2^ of herniation area at Time 1, a decrease of 41 mm^2^ and 50 mm^2^ of disc height could be expected after four and eight years, respectively.

**Table 4:** Cross-sectional and longitudinal associations between size of LDH, dural sac area and disc height

		β-coefficient (95% CI)	P-value
**LDH→Disc height**			
Cross-sectional		**0.353 (0.142; 0.564)**	0.001
Four-year change		**−0.413 (−0.603; −0.224)**	0.000
Eight-year change		**−0.504 (−0.807; −0.201)**	0.001
**LDH→Dural sac**			
Cross-sectional		−0.254 (−0.515; 0.007)	0.057
Four-year change		−0.130 (−0.262; 0.003)	0.055
Eight-year change		**−0.354 (−0.581; −0.128)**	0.003
**Disc height→Dural sac**			
Cross-sectional		**0.282 (0.158; 0.406)**	0.000
Four-year change		−0.041 (−0.102; 0.020)	0.189
Eight-year change		0.005 (−0.112; 0.122)	0.935
**LDH & disc height (multivariable regression) → Dural sac**			
Cross-sectional	LDH	**−0.364 (−0.621; −0.107)**	0.006
	Disc height	**0.312 (0.187; 0.436)**	0.000
Four-year change	LDH	−0.112 (−0.253; 0.028)	0.114
	Disc height	−0.240 (−0.088; 0.040)	0.462
Eight-year change	LDH	**−0.405 (−0.645; −0.165)**	0.001
	Disc height	0.072 (−0.045; 0.189)	0.222

Presented as regression coefficients for sizes of LDH with corresponding 95% confidence intervals, for both univariate and multiple linear regression. Significant associations are shown in bold

There was no statistically significant association between LDH and dural sac area in the cross-sectional or four-year longitudinal analyses. However, for the eight-year longitudinal analysis, large herniations predicted smaller dural sac areas with a decrease of 35 mm^2^ of dural sac area for each 100 mm^2^ of herniation area at Time 1 (*p* < 0.003, Table 4).

There was a statistically significant cross-sectional association between disc height and dural sac area. An increment of 100 mm^2^ in disc height area, was associated with an increase in the dural sac area of 28 mm^2^. No longitudinal associations were found (see Table 4).

In the multivariable linear regression analyses of cross-sectional associations, both LDH and disc height were statistically significantly associated with dural sac areas. Larger herniations and lower disc height were associated with smaller dural sac areas (Table 4).

For the longitudinal multiple regression analyses, only LDH was significantly negatively associated with eight-year changes in dural sac area. When the interaction term for disc height and LDH was taken into account, the interaction was significant, but coefficients were too small to have any impact (0.000; −0.006).

## Discussion

### Summary

To our knowledge, this is the first study to investigate changes in the size of LDHs, dural sacs and disc heights over multiple follow ups, using a reliable measurement method with high intra- and inter-observer agreement.

Our results confirmed the hypothesis that increased size of LDH would be associated with a decrease in size of the dural sac and the disc height over time. We also found, in the cross-sectional analyses, that larger LDH was correlated with smaller dural sac area and larger disc height.

### Comparison with the literature

Adams and Dolan have suggested an ‘annulus-driven’ type of disc degeneration, with LDH being a possible endpoint with migration of the nucleus through radial fissures into the annulus [20]. We based our hypothesis on this postulation because this type of disc degeneration is common in the lower lumbar spine and becomes more common during ageing [21, 22]. However, uncertainty still remains about LDH being the cause of, consequence of, or just a part of, the degenerative pathway for lumbar discs [23].

The current study showed that the majority of LDHs do not change in size over time, which is contrary to previous literature [2, 3, 24–26]. This may be the result of a different length in follow-up times and/or different methodologies used to measure the changes. Furthermore, we were conservative in defining changes, as they were based on the minimal detectable change and therefore might not be due to measurement error. Lastly, we studied a general population rather than a patient population.

Longitudinal studies have shown that disc height decreases over time [6, 27, 28], but these results are not directly comparable to ours because we only studied disc height in relation to people with defined LDH.

In the current study, we found a positive correlation between LDH and disc height measured at the same time-point. This is in contrast to one study of patients with LDH which reported no significant association between size of LDH and disc height [7]. However, our results are in line with those that have been observed in another study of patients with severe LBP undergoing discography, where the authors reported a moderate positive correlation between LDH and disc height [4]. If there truly is an association between the two measures, the reason for this may simply be that discs with larger height produce larger herniations due to a larger overall volume.

In the literature, there have been cross-sectional studies reporting associations between LDH or disc degeneration with spinal canal area or dural sac area [29, 30]. In these studies, the dural sac areas were smaller in patients having discectomy, compared with controls, which are comparable with our results. In relation to dural sac area, one study reported a decrease in dural sac area over time for patients with lumbar spinal stenosis [31].

### Strengths & limitations

This study has several strengths. The cohort was the same age and assembled from the general population. We used a known and validated method for evaluating LDH from MRI [14] and a validated quantitative measuring method for calculating the sizes of LDH, dural sac and disc height [15]. The raters were blinded to any clinical information about the participants and to each other’s assessments. Defining the changes in size over time was based on LOA, which are a clinically relevant metric [15]. Lastly, we had two follow ups equally spaced over an eight-year period.

The study has some limitations. The decision to measure only disc levels from the time-point where LDH was detected, based on the ‘Combined task force’ classification [13] was determined by our aim of studying primarily discs with LDH. Measurements were not taken of discs without LDH and therefore our results cannot be generalizable to these discs. Measurement of LDH would not be meaningful in the full study sample, but measurements of changes in disc height and dural sac in those without LDH would have made it possible to make interesting comparisons to the potential extra height reduction caused by LDH.

### Research implications

Our study is the first step on the path to describing changes in LDH, dural sac and dural sac over time. However, as we have only used disc levels with LDH, the next step will be to include discs without LDH in order to determine if the changes are the result of normal wear and tear or due to the LDH itself. In future investigations, links between different degenerative pathways with or without LDH may make it possible to study their relevance for pain and disability in patients.

## Conclusion

We conclude that, on average, most lumbar disc herniations do not change significantly over a period of four to eight years. As hypothesised, larger herniation size predicted a reduction in both dural sac area and disc height over a four- to eight-year period. It is unknown whether a reduction in dural sac area and disc height over time is caused by the LDH or other factors. Further research would be useful to determine the correlations between the progression of LDH and resolution of patient symptoms.

## Abbreviations

AT: Andreas Tunset
BSc: Bachelor of Science
CI: Confidence interval
CSA: Cross-sectional area
CSV: Comma separated values
EB: Eleanor Boyle
ID: Identification
LDH: Lumbar disc herniation
LOA: Limits of agreement
mm^2^: Square millimetres
MRI: Magnetic resonance imaging
MSc: Master of Science
n: Number
PhD: Doctor of Philosophy
PK: Per Kjaer
PT: Physical therapist
ROI: Region of interest
T: Tesla
TSJ: Tue Secher Jensen
